# Trends in Severe Maternal Morbidity, Obstetric Comorbidities, and Birth Complications in Illinois

**DOI:** 10.1097/og9.0000000000000046

**Published:** 2024-11-21

**Authors:** Mugdha Mokashi, Lynn Yee, Joseph Feinglass

**Affiliations:** Department of Obstetrics and Gynecology, the Division of Maternal-Fetal Medicine, Department of Obstetrics and Gynecology, and the Division of General Internal Medicine and Geriatrics, Northwestern University Feinberg School of Medicine, Chicago, Illinois.

## Abstract

**OBJECTIVE:**

To analyze trends in severe maternal morbidity (SMM) and route-specific complications of birth in Illinois hospitals from January 2016 to June 2023.

**METHODS:**

This retrospective population-based cohort study analyzed birth admission data from 159 hospitals using the Illinois Hospital Association Comparative Health Care and Hospital Data Reporting Services database from January 2016 to June 2023. Primary outcomes were incidence of SMM and route-specific delivery complications. Secondary outcomes included incidence of chronic and pregnancy-related comorbidities. Poisson regression was used to estimate incidence rate ratios for SMM; logistic regression was used to estimate odds ratios for route-specific birth complications; and model SEs were adjusted for clustering of observations within hospitals.

**RESULTS:**

In this cohort of 988,480 births, the overall SMM rate was 1.6%; the rate of SMM rose from 1.4% in 2016 to 2.0% in 2023. The rate of SMM was 0.9% for people who underwent vaginal births and 3.2% for people who underwent for cesarean births. Vaginal birth complications (7.3% overall) increased 22.4%, and cesarean birth complications (10.9% overall) increased 48.9%. Hypertensive disorders of pregnancy and anemia, both of which increased over the study period, were significant risk factors for SMM and birth complications. Over the study period, there were also increases in International Classification of Diseases, Tenth Revision–coded gestational diabetes (4.2–5.5%), depression (2.5–6.6%), anxiety (3.1–10.4%), and other chronic comorbidities (4.7–7.4%). Non-Hispanic Black patients had more than double the SMM rate (2.6%) compared with non-Hispanic White patients (1.1%).

**CONCLUSION:**

From 2016 to 2023, the rates of SMM and birth complications in Illinois increased alongside increases in coding for chronic and prepregnancy and pregnancy-related comorbidities. This study demonstrates the burden of cesarean birth–associated adverse outcomes, including a higher rate of SMM, which increased over the study period.

## Abstract 2

From 2016 to 2023, the rates of severe maternal morbidity and birth complications in Illinois increased alongside increases in chronic and pregnancy-related comorbidities.

Severe maternal morbidity (SMM) and other birth and postpartum complications have increased over time both nationally and in Illinois.^1,2^ Worsening maternal complications can be attributed to many factors, including the increase in the age of those giving birth, combined with the increasing prevalence of conditions such as obesity, hypertensive disorders of pregnancy (HDP), and gestational diabetes, among pregnant and postpartum individuals of all ages.^3–7^ Large racial disparities in SMM have been documented nationally and in Illinois; in 2020, Black individuals were found to have double the rate of SMM and triple the maternal mortality rate compared with White individuals within the state.^2^

In 2021, Oot et al^8^ published birth outcomes at Illinois hospitals in 2016–2017, finding that the Illinois SMM rate was 1.4% overall, 0.4% for people who had vaginal births, and 2.8% for people who had cesarean births. They also reported composite birth complication rates of 6.9% and 10% for vaginal and cesarean births, respectively.^8^ Since 2017, there have been improvements in prepregnancy and postpartum insurance coverage, with more than 1 million additional Illinois residents having Medicaid insurance in 2023 compared with 2013^9^ and extension of Illinois Medicaid postpartum coverage in 2021.^10^ Ongoing state-level quality improvement efforts are targeting racial disparities and birth outcomes.^11,12^ Thus, using the same methods as Oot et al, we aim to analyze trends over subsequent years in SMM and birth route–specific complications. We also analyze the associations of sociodemographic and clinical characteristics with birth outcomes to evaluate outcomes among the patient populations at highest risk.

## METHODS

This is a population-level retrospective cohort study using administrative hospital data from the Illinois Hospital Association Comparative Health Care and Hospital Data Reporting Services database. We analyzed all birth admissions from January 2016 to June 2023. Hospital records included patient demographic characteristics, insurance status, residential ZIP code, and International Classification of Diseases, Tenth Revision (ICD-10) diagnosis and procedure codes.

Severe maternal morbidity was identified by Centers for Disease Control and Prevention criteria using SMM-associated ICD-10 codes comprising a list of 21 complications, including transfusions, as well as a nontransfusion rate.^13^ Route-specific complications were identified with an algorithm created by Asch et al,^14^ which was used to measure quality of care for approximately 5 million births in Florida and New York with International Classification of Diseases, Ninth Revision coding. We updated the Asch et al algorithm to identify infection, thrombosis, or hemorrhage-related diagnoses for vaginal birth complications in addition to operative complications for cesarean birth complications using ICD-10 coding.^8,14^ For birth complications, we omitted obstetric laceration–related complications in line with American College of Obstetricians and Gynecologists recommendations.^15^

Maternal sociodemographic characteristics included maternal age at time of birth and reported race and ethnicity characterized as Asian, Hispanic, non-Hispanic Black, non-Hispanic White, and none of the above or unknown (which include multiracial or American Indian/Alaskan Native). Race and ethnicity were analyzed as a variable in this study to describe potential disparities in maternal birth outcomes. Insurance status was categorized as Medicaid or non-Medicaid, the latter of which included private insurance or other insurance, including Medicare. Patient county of residence as determined by ZIP code was separated into three regions: Cook County (which includes the city of Chicago), the five surrounding suburban “collar” counties (DuPage, Kane, Lake, McHenry, and Will), or any other Illinois “downstate” counties. Patient residence ZIP codes were also matched to ZIP Code Tabulation Areas using the American Community Survey 2021 5-year Census data to determine the percent of families in that ZIP code living at or below the federal poverty level. Patients from non-Illinois ZIP codes were categorized as non-Illinois residents.

Pregnancy clinical characteristics included route of birth (cesarean or vaginal), single or multifetal gestation, and history of previous cesarean birth. We used the ICD-10 coding from the Leonard et al^16^ model of SMM incidence to identify clinical obstetric comorbidities, including chronic hypertension, HDP (including gestational hypertension, preeclampsia, and eclampsia), gestational diabetes, pregestational diabetes mellitus, anemia, and asthma. In addition to these discrete obstetric comorbidities, we created a single indicator for any of several less common but potentially serious comorbid conditions, including pulmonary hypertension, hematologic disorder, chronic heart disease, chronic renal disease, connective tissue disorder, history of bariatric surgery, neuromuscular disease, thyroid disorder, human immunodeficiency virus (HIV), acquired immunodeficiency syndrome (AIDS), and chronic liver disease. We used the presence of an ICD-10 obesity code as a risk factor flag. The ICD-10–coded mental health conditions included depression, anxiety, and serious mental illness (schizophrenia, bipolar disorder, personality disorder, hallucinations, or other psychosis). *Substance use* was defined as any ICD-10 diagnosis for dependence or abuse of alcohol, opioids, cannabinoids, sedatives, cocaine, stimulants, hallucinogens, tobacco, or volatile solvents.

We used χ^2^ tests to determine the significance of differences in SMM and complication incidence by year of delivery and patient characteristics. Poisson regression, which provides a better estimate of relative risk for low-incidence outcomes, was used to estimate incidence rate ratios for SMM.^17^ Logistic regression was used to estimate odds ratios for route-specific complications. Regression model SEs were adjusted for clustering of observations within hospitals. There were no missing data for year, age, or insurance status. Missing data within the category of race and ethnicity were included in the none of the above or unknown category. Admissions with missing ZIP code data (n=63) were classified as non-Illinois residents. Finally, all other missing data were addressed with dummy variable adjustment. Analyses were performed with IBM SPSS 29 and Stata 17. The study data set comprised publicly available, deidentified data and was deemed exempt by the Northwestern University IRB.

## RESULTS

There were 988,480 births at 159 Illinois hospitals over the study period, including 686,232 (69.4%) vaginal births and 302,248 (30.6%) cesarean births. The overall rate of SMM was 1.6%, with a 0.9% rate of SMM for people who underwent vaginal births and 3.2% for people who underwent cesarean births. The vaginal birth complication rate was 7.3%, and the cesarean birth complication rate was 10.9%. There were 40 in-hospital maternal deaths.

**Fig. 1.**
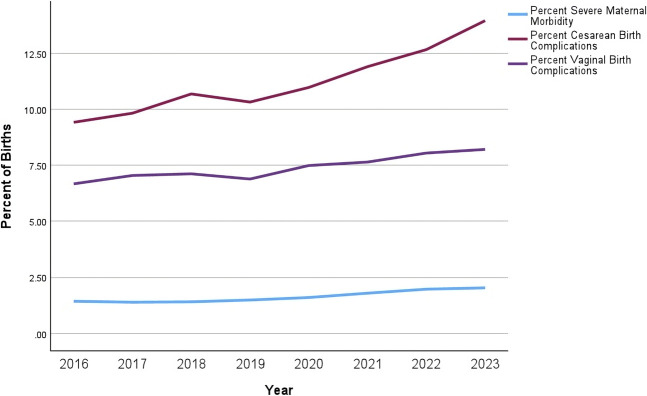
Annual trends in severe maternal morbidity (SMM) and route-specific birth complications in Illinois from 2016 through the first 6 months of 2023. N=988,480 births at 127 Illinois hospitals, January 2016–June 2023. All annual comparisons *P*<.001. Births with SMM are identified by the Centers for Disease Control and Prevention–defined 21 indicators of SMM and the associated International Classification of Diseases, Tenth Revision (ICD-10) codes. Cesarean birth complications include ICD-10 codes associated with operative complications in addition to infection, thrombosis, or hemorrhage-related diagnoses associated with the birth hospitalization. Vaginal birth complications include infection, thrombosis, and hemorrhage-related diagnoses associated with the birth hospitalization.

Figure 1 presents trends in birth outcomes over the 7.5 years from 2016 to 2023. The annual rate of SMM rose from 1.4% in 2016 to 2.0% in the first 6 months of 2023. Annual vaginal birth complications increased by 22.4% and cesarean complications increased by 48.9% from 2016 through the first 6 months of 2023.

*Mokashi. Trends in Illinois Birth Complications and SMM. O&G Open 2024*.

**Fig. 2.**
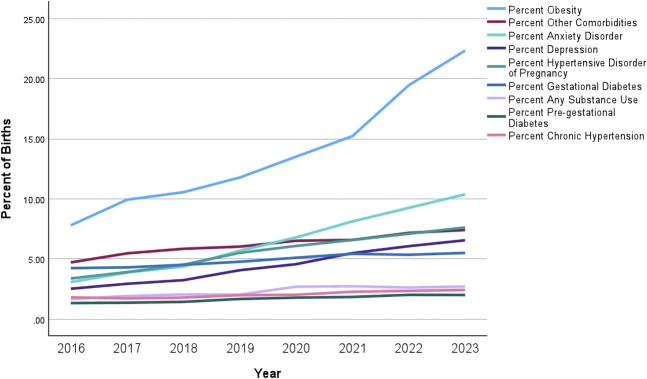
Annual trends in maternal comorbidities in Illinois births from January 2016 through the first 6 months of 2023. N=988,480 births at 127 Illinois hospitals, January 2016–June 2023. All annual comparisons *P*<.001. Hypertensive disorders of pregnancy include gestational hypertension, preeclampsia, eclampsia, and superimposed preeclampsia. Selected other comorbidities include pulmonary hypertension, hematologic disorder, chronic heart disease, chronic renal disease, connective tissue disorder, history of bariatric surgery, neuromuscular disease, thyroid disorder, human immunodeficiency virus (HIV), acquired immunodeficiency syndrome (AIDS), and chronic liver disease.

Figure 2 shows annual trends in ICD-10–coded obstetric comorbidities. There were large increases from 2016 through the first 6 months of 2023 in monthly rates of coded gestational diabetes (4.2% to 5.5%), HDP (3.4% to 7.6%), anemia (9.9% to 17.6%), depression (2.5% to 6.6%), anxiety (3.1% to 10.4%), and other chronic comorbidities (4.7% to 7.4%). Rates of codes for pregestational diabetes, chronic hypertension, and substance use were relatively stable over the study period. There was a particularly large increase in annual rates of ICD-10–coded obesity from 2016 through the first 6 months of 2023 (7.8% to 22.3%).

*Mokashi. Trends in Illinois Birth Complications and SMM. O&G Open 2024*.

Table 1 presents SMM and route-specific complication rates by patient sociodemographic and clinical characteristics for the study period, with both bivariate and regression model results presented for all three birth outcomes. All bivariate comparisons for all three outcomes (SMM, cesarean birth complications, and vaginal birth complications) were statistically significant (*P*<.001). The annual change in SMM was not significant in the adjusted regression model, whereas regression results for route-specific complication outcomes did demonstrate a statistically significant increase over time. Bivariate results comparing 2016 with the first 6 months of 2023 show a 42.9% increase in SMM, a 22.4% increase in vaginal birth complications, and a 48.9% in cesarean birth complications. The regression results for year of birth show a nonsignificant 11.0% increase in the likelihood of SMM from 2016 through the first 6 months of 2023, but there was a significantly higher likelihood of cesarean birth complications by 2021, with a 41.7% higher likelihood in 2023. Compared with 2016, vaginal birth complications had a 11.8% higher likelihood in 2023.

**Table 1.** Incidence of Severe Maternal Morbidity and Route-Specific Birth Complications by Patient Sociodemographic and Clinical Characteristics in Illinois Hospitals (n=159) From January 2016 to June 2023

Characteristic	% of All Births (n=988,480)	Rate of SMM (n=15,822)*	IRR (95% CI)	Cesarean Birth Complication Rate (n=302,248)†	aOR (95% CI)	Vaginal Birth Complication Rate (n=686,232)†	aOR (95% CI)
Year							
2016	14.4	1.4	Ref	9.4	Ref	6.7	Ref
2017	14.4	1.4	0.91 (0.83–0.99)	9.8	1.03 (0.96–1.10)	7.0	1.04 (0.98–1.11)
2018	13.9	1.4	0.89 (0.80–0.99)	10.7	1.12 (1.01–1.25)	7.1	1.05 (0.96–1.14)
2019	13.5	1.5	0.91 (0.79–1.05)	10.3	1.06 (0.96–1.17)	6.9	1.00 (0.92–1.08)
2020	12.8	1.6	0.93 (0.81–1.24)	11.0	1.12 (0.98–1.27)	7.5	1.07 (0.98–1.17)
2021	12.7	1.8	1.03 (0.86–1.24)	11.9	1.21 (1.05–1.39)	7.6	1.07 (0.98–1.16)
2022	12.3	2.0	1.09 (0.90–1.32)	12.6	1.28 (1.10–1.51)	8.0	1.11 (1.02–1.21)
2023	5.8	2.0	1.11 (0.95–1.30)	14.0	1.42 (1.21–1.66)	8.2	1.12 (1.02–1.23)
Sociodemographic characteristics							
Age (y)							
Younger than 19	2.3	2.3	1.67 (1.53–1.82)	14.7	1.48 (1.30–1.68)	9.3	1.36 (1.28–1.45)
19–24	18.4	1.7	1.20 (1.15–1.25)	11.8	1.16 (1.11–1.22)	7.7	1.11 (1.07–1.15)
25–29	26.9	1.5	Ref	10.2	Ref	7.0	Ref
30–34	31.7	1.4	0.95 (0.89–1.01)	10.5	1.00 (0.95–1.04)	7.1	0.98 (0.95–1.02)
35–39	17.0	1.7	1.01 (0.93–1.11)	11.1	0.99 (0.94–1.04)	7.1	0.92 (0.89–0.95)
Older than 40	3.8	2.6	1.23 (1.11–1.35)	13.3	1.15 (1.06–1.23)	8.5	1.06 (1.00–1.13)
Race and ethnicity							
Asian	5.0	1.6	1.44 (1.25–1.66)	14.6	1.59 (1.44–1.76)	9.8	1.56 (1.44–1.70)
Hispanic	18.5	1.8	1.39 (1.22–1.59)	13.1	1.28 (1.18–1.41)	9.0	1.33 (1.25–1.42)
Non-Hispanic Black	15.8	2.6	1.34 (1.19–1.53)	13.8	1.17 (1.04–1.32)	8.2	1.03 (0.95–1.12)
Non-Hispanic White	50.4	1.1	Ref	8.7	Ref	6.0	Ref
None of the above or unknown	10.3	1.9	1.42 (1.22–1.64)	12.2	1.26 (1.11–1.45)	7.9	1.20 (1.10–1.31)
Insurance status							
Medicaid	36.9	2.0	1.12 (1.02–1.24)	11.0	0.87 (0.80–0.94)	7.4	0.89 (0.84–0.93)
Private, uninsured, other	63.1	1.4	Ref	10.9	Ref	7.2	Ref
County							
Cook	42.3	1.9	Ref	15.7	Ref	9.4	Ref
Collar counties‡	24.4	1.3	0.86 (0.66–1.11)	7.7	0.50 (0.42–0.60)	5.5	0.63 (0.55–0.71)
Downstate counties‡	33.3	1.4	0.84 (0.62–1.15)	7.8	0.50 (0.39–0.64)	5.8	0.66 (0.57–0.78)
State of residence							
Illinois resident	98.7	1.6	Ref	10.9	Ref	7.3	Ref
Non-Illinois resident	1.3	1.9	1.31 (1.05–1.64)	12.4	1.62 (1.23–2.13)	6.0	1.00 (0.84–1.18)
% households below poverty level in ZIP code§							
Less than 5	32.4	1.3	Ref	9.5	Ref	6.5	Ref
5–10	30.4	1.4	0.98 (0.91–1.06)	10.7	1.05 (0.99–1.11)	7.3	1.03 (0.99–1.08)
10–20	27.8	1.9	1.15 (1.04–1.28)	11.6	1.05 (1.00–1.20)	7.6	1.03 (0.96–1.10)
More than 20	8.2	2.8	1.29 (1.10–1.51)	15.4	1.21 (1.03–1.43)	9.4	1.11 (1.01–1.23)
Pregnancy-related factors							
Type of pregnancy							
Singleton	98.2	1.5	Ref	10.7	Ref	7.2	Ref
Multifetal gestation	1.8	5.5	1.76 (1.57–1.97)	16.8	1.59 (1.45–1.75)	19.8	2.74 (2.52–2.98)
Birth type							
Vaginal	69.4	0.9	Ref	—	—	—	—
Cesarean	30.6	3.2	2.75 (2.52–3.00)	—	—	—	—
Previous cesarean	5.4	4.0	1.34 (1.23–1.47)	11.1	0.97 (0.92–1.01)	11.2	1.43 (1.34–1.53)
Pregnancy complications							
Gestational diabetes	4.8	2.4	1.01 (0.93–1.09)	10.4	0.88 (0.83–0.94)	9.7	1.19 (1.13–1.26)
HDP‖	5.4	4.6	1.77 (1.65–1.90)	17.6	1.52 (1.43–1.61)	14.8	1.82 (1.73–1.91)
Clinical comorbidities							
Pregestational diabetes	1.6	4.1	1.22 (1.11–1.33)	13.0	1.03 (0.92–1.15)	10.9	1.19 (1.08–1.31)
Chronic hypertension	2.0	5.3	1.31 (1.22–1.40)	12.8	0.98 (0.92–1.04)	12.5	1.34 (1.24–1.46)
Anemia	13.1	4.1	2.08 (1.85–2.34)	15.7	1.45 (1.37–1.53)	11.6	1.57 (1.43–1.73)
Asthma	5.4	4.4	1.65 (1.49–1.83)	15.2	1.20 (1.13–1.27)	10.3	1.22 (1.16–1.28)
Obesity	13.0	2.4	0.83 (0.75–0.95)	13.3	1.15 (1.06–1.26)	10.2	1.28 (1.20–1.35)
Serious mental illness	0.9	2.8	0.85 (0.75–0.95)	12.7	1.01 (0.90–1.14)	7.7	0.92 (0.82–1.04)
Depression	4.2	2.4	0.92 (0.84–1.00)	12.9	1.05 (0.98–1.12)	8.4	0.98 (0.94–1.03)
Anxiety	6.0	2.6	1.16 (1.08–1.25)	13.2	1.17 (1.11–1.23)	8.9	1.19 (1.13–1.25)
Any substance use	2.3	3.0	1.32 (1.18–1.47)	11.5	1.08 (0.98–1.18)	7.1	0.97 (0.89–1.05)
Other comorbidity¶	6.1	8.1	4.71 (3.99–5.57)	16.7	1.46 (1.38–1.55)	11.3	1.44 (1.35–1.52)

SMM, severe maternal morbidity; IRR, incidence rate ratio; aOR, adjusted odds ratio; Ref, referent; HDP, hypertensive disorder of pregnancy.

Data are n (%) unless otherwise specified.

Regression results are fully adjusted for all covariates.

*P*<.01 for all bivariate comparisons except for the following categories and their respective *P* values: for SMM: age: 30–34 years (.11), 35–39 years (.74); Medicaid insurance (.02); county: collar counties (.25), downstate counties (.29); 5–10% below poverty level in ZIP code (.65); gestational diabetes (.81); depression (.06); for cesarean birth complications: age: 30–34 years (.88), 35–39 years (.76); below poverty level in ZIP code: 5–10% (.12), 10–20% (.06), more than 20% (.02); previous cesarean birth (.14); clinical comorbidities: preexisting diabetes mellitus (.58), chronic hypertension (.44), serious mental illness (.81), depression (.15), any substance use (.13); for vaginal birth complications: age: 30–34 years (.35), age 40 years or older (.07); non-Hispanic Black race (.42); non-Illinois resident (.95); below poverty level in ZIP code: 5–10% (.14), 10–20% (.44), more than 20% (.04): clinical comorbidities: serious mental illness (.18), depression (.41), any substance use (.40).

* Births with SMM are those coded by for Centers for Disease Control and Prevention–defined 21 indicators of SMM and associated International Classification of Diseases, Tenth Revision (ICD-10) codes.

† Cesarean birth complications include ICD-10 codes associated with operative complications in addition to infection, thrombosis, or hemorrhage-related diagnoses associated with the birth hospitalization. Vaginal birth complications include infection, thrombosis, or hemorrhage-related diagnoses associated with birth hospitalization.

‡ Collar counties refer to DuPage, Kane, Lake, McHenry, and Will Counties. Downstate counties are all other Illinois counties excluding Cook County and collar counties.

§ This category reflects the percentage of residents living in the patient's identified ZIP code of residence who were at or below the Federal Poverty Level. Patient ZIP codes were matched to Zip Code Tabulation Areas using the American Community Survey 2021 5-year Census data.

‖ Includes gestational hypertension, preeclampsia, eclampsia, and superimposed preeclampsia.

¶ Includes pulmonary hypertension, hematologic disorder, chronic heart disease, chronic renal disease, connective tissue disorder, history of bariatric surgery, neuromuscular disease, thyroid disorder, human immunodeficiency virus (HIV), acquired immunodeficiency syndrome (AIDS), and chronic liver disease.

In addition to year of birth, other demographic factors were associated with SMM and route-specific complications, including extremes of age (Table 1). Non-Hispanic Black individuals had the highest rate of SMM (2.6%) and, after adjustment for clinical covariates, were 34.6% more likely to experience SMM compared with non-Hispanic White individuals (1.1%). Having Medicaid insurance was associated with higher rates of SMM and route-specific complications. Route-specific complications were more pronounced in Cook County compared with other counties of residence in Illinois. Zip Code Tabulation Area poverty level was associated with rates of SMM and route-specific complications, with the highest rates for those living in ZIP codes where more than 20% of households were at or below the poverty level.

People with multifetal gestation had a much higher rate of SMM (5.5%, incidence rate ratio 1.76) than those with singleton gestations (1.5%) (Table 1). Route-specific complications were also higher among people with multifetal gestation. After adjustment, people with cesarean births had a nearly threefold increased risk of SMM (3.2%, incidence rate ratio 2.75) compared with people with vaginal births (0.9%). Hypertensive disorders of pregnancy (4.6%, incidence rate ratio 1.77) and anemia (4.1%, incidence rate ratio 2.08) were among the most significant risk factors for SMM. Prepregnancy diabetes, chronic hypertension, asthma, anxiety, and any substance use also conferred increased risk for SMM. Having an ICD-10 obesity code conferred additional risk for both vaginal and cesarean birth complications but not SMM risk. Those with other comorbidities made up 6.1% of the sample and had a nearly fivefold greater risk of SMM, in addition to significantly increased risk for both cesarean and vaginal birth complications.

## DISCUSSION

In this update on Illinois birth outcomes, we demonstrate rising rates of SMM and route-specific birth complications, as well as prepregnancy and pregnancy-related health conditions, from 2016 to 2023. Despite significant recent statewide quality improvement efforts, these birth outcomes are worsening for all ages, reflecting the worsening prepregnancy health of the reproductive-age population in Illinois.

The prior Illinois study by Oot et al^8^ reported an SMM rate of 1.4% from 2016 to 2018, which we found rose to 1.6% over the 90-month period from 2016 to 2023. We found slightly higher birth complication rates, with the average cesarean birth complication rate at 10.9% compared with 10.0% reported by Oot et al^8^ and the average vaginal birth complication rate at 7.3% compared with the previously reported 6.9%. We present an SMM rate based on coded transfusion procedures, which may be sensitive to hospital procedure coding. We also found that the nontransfusion SMM rate shows the same trend, rising continuously every year from 0.8% in 2016 to 1.0% in 2023. This 25.0% increase in the nontransfusion SMM rate was proportionally lower than the 42.9% increase in the transfusion inclusive SMM rate over the same period.

Consistent with prior research in Illinois^2^ and nationally, we found racial and economic disparities in both SMM and birth complications. We also identified that residence in a high-poverty-level ZIP code was associated with a risk of SMM and birth complications independently of race and ethnicity. Given that poverty disproportionately affects minoritized populations, our findings highlight the intersectional nature of social determinants of health.

We found that ICD-10 coding of maternal chronic conditions has remained stable or, for some conditions, has increased over the study period. There was a dramatic increase in coding for obesity, which includes ICD-10 diagnoses of pregnancy-specific obesity and body mass index (BMI, calculated as weight in kilograms divided by height in meters squared) higher than 30. The sharp increase in obesity coding likely does not reflect the epidemiology of obesity in this population; the hospital-coded prevalence was only 13.0%, which is likely an underestimate given data finding that one in three Illinois residents lives with obesity.^18^

The increased prevalence of comorbid conditions does not appear to be simply attributable to the aging of the birthing population. The proportion of births for teens fell from 2.8% in 2016 to 1.8% in the beginning of 2023, consistent with national data suggesting a declining adolescent birth rate.^19^ Conversely, from 2016 to 2023, births among those aged 35–39 years increased from 15.0% to 18.4%, and births among those aged 40 year or older increased from 3.2% to 4.4% (data not shown). However, the increase in comorbid conditions also occurred for younger individuals over this period. For example, among births to those younger than 30, coding increased for HDP (3.1% to 7.7%), anemia (11.1% to 20.2%), gestational diabetes (2.6% to 3.5%), other chronic comorbidities (4.0% to 6.3%), depression (2.5% to 6.9%), and serious mental illness (0.9% to 1.5%) (data not shown). This suggests that an overall deterioration in the health of the reproductive-age population is driving adverse birth outcomes in Illinois rather than solely increasing maternal age.

Understanding the mechanisms behind rising rates of adverse outcomes is crucial for improving maternal health. A key focus in primary prevention of maternal morbidity is addressing chronic stress. Exposure to traumatic events and lifetime composite stress burden^20^ have been linked with development of HDP and perinatal mood disorders.^21^ People who identified as women with multiple adverse childhood events are more likely to have diagnoses of obesity, hypertension, and diabetes than those without these events.^22^ Chronic stress is also exacerbated by racism and poverty, resulting in higher rates of pregnancy-related morbidity, known as weathering.^21,23^ Poverty alleviation policies have been shown to improve maternal health.^24,25^ In Illinois, the proposed fully refundable child tax credit in Illinois House Bill 4917 could benefit maternal health, although it has not yet been passed.^26^ Linking maternal morbidity and mortality prevention to economic justice initiatives addresses the upstream causes of some of these comorbidities.

Another strategy to improve obstetric outcomes is through health systems improvement, particularly through statewide perinatal quality initiatives. In California, postpartum hemorrhage–related SMM was reduced in hospitals participating in a statewide collaborative compared with nonparticipating hospitals in the state.^27^ The Illinois Perinatal Quality Collaborative currently targets safe reduction of cesarean births through the Promoting Vaginal Birth quality improvement initiative,^28^ in addition to addressing racial disparities through the Illinois Perinatal Quality Collaborative Birth Equity initiative.^11^ The extent to which these policies bend the curve of adverse birth outcomes in Illinois remains to be determined.

Local initiatives targeting direct patient support may also meaningfully affect maternal morbidity. Doula support during labor is associated with a reduction in cesarean birth rate.^29^ In 2024, the Illinois Department of Healthcare and Family Services changed reimbursement coverage of doula services with the aim of increasing access to doulas.^30^ Patient navigator programs ongoing in Illinois have been associated with reduced disparities in care utilization among minoritized patients with low income in the postpartum period.^31,32^

There are several limitations to this study. Birth hospitalization discharge data used in this study are not linked to neonatal or postpartum outcomes and are limited in characterizing longitudinal health outcomes. We report only maternal deaths defined as discharge diagnoses, which underestimates the true burden of maternal mortality compared with 1-year postpartum estimates.^2^ It is likely that ICD-10 coding underestimates the actual rate of several maternal conditions in this data set, including obesity and other chronic conditions. This may be attributable, in part, to disparities in care utilization, which affects patients' ability to receive diagnoses. Furthermore, coding data quality is limited by hospital medical record coding capacity. Coding intensity varies by hospital birth volume,^33^ which can result in bias in complications or the prevalence of comorbidities. However, it is unknown whether this would affect the changes over time in the complication rate and disease prevalence demonstrated in this study.^8^

Overall, the size and diversity of this data set provide important longitudinal data on targetable chronic disease burden and drivers of SMM. More broadly, change at the social, economic, and political levels is necessary to shift birth outcomes. At the federal level, passage of the 13 bills making up the Black Maternal Health Momnibus Act reintroduced in 2023 would provide critical funding support to increase data collection and quality initiatives to prevent maternal morbidity.^34^ The NIH Implementing a Maternal Health and Pregnancy Outcomes Vision for Everyone initiative, launched in 2019, awards research funding to reduce preventable causes of maternal mortality and health disparities in the peripartum period.^35^ The lifelong consequences on maternal health after adverse pregnancy outcomes^36^ reflect the urgent need for optimizing health in this critical life stage.
